# The Association of Serum Calprotectin with Fitness Indicators and Biochemical Markers in High-Level Athletes: A Continuous Dynamic Monitoring during One Competitive Season

**DOI:** 10.3390/sports11120243

**Published:** 2023-12-14

**Authors:** Frane Bukvić, Alan Ivković, Helena Čičak, Lora Dukić, Ana-Maria Šimundić, Domagoj Marijančević, Daria Pašalić

**Affiliations:** 1 Department of Orthopedics and Trauma Surgery, University Hospital ‘Sveti Duh’, 10000 Zagreb, Croatia; frane.bukvic@gmail.com (F.B.); alan.ivkovic@gmail.com (A.I.); 2 School of Medicine, University of Zagreb, 10000 Zagreb, Croatia; 3 Department of Medical Laboratory Diagnostics, University Hospital ‘Sveti Duh’, 10000 Zagreb, Croatia; helena.cicak5@gmail.com (H.Č.); lora.dukic@gmail.com (L.D.); am.simundic@gmail.com (A.-M.Š.); 4 Faculty of Pharmacy and Biochemistry, University of Zagreb, 10000 Zagreb, Croatia; 5 Department of Clinical Chemistry, University Hospital Centre ‘Sestre Milosrdnice’, 10000 Zagreb, Croatia; domagoj.marijancevic@kbcsm.hr; 6 Department of Medical Chemistry, Biochemistry and Clinical Chemistry, School of Medicine, University of Zagreb, 10000 Zagreb, Croatia

**Keywords:** calprotectin, physical fitness, biochemical markers, water polo players, dynamic monitoring

## Abstract

The objective was to determine the associations between several biochemical indicators and the dynamics of concentration change across four physical fitness phases over the period of a competitive season. Furthermore, associations between serum calprotectin and biomarkers of inflammation or muscle injury and physical indicators were examined. Subjects and methods: Twenty professional male water polo players (median age: 21 (15–31)) were included in this study. Serum creatine kinase activity was determined by the automated photometric UV method. The concentrations of calprotectin, C-reactive protein, and myoglobin were measured using an automated immunoturbidimetric method, while an automated immunochemistry method was employed for interleukin-6, troponin I, and cortisol determination. Tests of repeated strength, maximal strength, and static strength were used to evaluate physical activity. Results: Serum calprotectin concentrations expressed in median and IQR were significantly different: T1: 2.92 g/mL (2.47; 3.86); T2: 2.35 g/mL (1.26; 2.87); T3: 2.27 g/mL (1.60; 3.27); and T4: 1.47 g/mL (1.04; 2.85) (*p* = 0.004). Cortisol concentration and CK activity showed significant changes among phases (*p* = 0.049 and *p* = 0.014, respectively). Each physical activity examined showed a significant seasonal decrease (all *p* values were 0.001). Calprotectin serum concentration and indicators of muscular injury, inflammation, and physical activity were found to be correlated during particular stages of the seasonal examination. Conclusions: Calprotectin values determined throughout one competitive season decreased as training intensity among water polo players increased. Serum calprotectin concentrations and indicators were related to biochemical markers of inflammation and muscle damage.

## 1. Introduction

Water polo is the longest-standing team sport at the Olympic Games [1], and it consists of intensive swimming, throwing, and wrestling [2]. Water polo players are extremely susceptible to various types of injuries, from traumatic injuries due to contact between players to overuse syndrome due to intensive training with repetitive throwing, swimming, and hitting movements [3]. Overuse injuries constitute 27.4% to 37.5% of all injuries in water sports, including water polo, with common issues such as shoulder tendinosis and muscle cramps in the lumbar spine [4]. Professional athletes undergo intense training to improve their performance [5]. Intensive training periods are combined with periods of rest in order to achieve the best possible adaptation of the organism to stress [6]. For this reason, great efforts are made to objectively quantify the relationship between training intensity and training tolerance [7]. Advances in technology allow us to determine the balance status between the training and recovery of professional athletes by using various intrinsic factors such as biochemical and hematological markers [8]. In order to assess the status of physical fitness better, measuring one biochemical marker is not enough, so several biochemical markers should be measured, and these results should be compared with certain indicators of physical fitness [9,10].

Biochemical markers of muscle injury (e.g., creatine kinase (CK), myoglobin, etc.) and their relationships with exercise have been widely studied [11,12,13]. CK peaks approximately 24 h after muscle-damaging exercise and can even stay elevated for 7 days, while myoglobin peaks approximately 1–3 h after exercise and returns to normal values 24 h after it [8,14]. Troponin values also rise after exercise, with their peak approximately 4–6 h after exercise, and return to normal values 24–48 h after it [14,15,16]. When markers of muscle damage are released into circulation, immune cells migrate to the site of injury to begin the process of repairing damaged tissue, releasing numerous cytokines and other signaling molecules to promote the inflammatory process [8]. Some studies showed that interleukin-6 (IL-6) concentrations increase after acute exercise and training [17,18]. The level of physical fitness of athletes is negatively correlated with the values of IL-6 [19]. Sedentary, low, moderate, and high levels of physical activity were classified based on the amount of calories burned and the type and intensity of exercise [19]. C-reactive protein (CRP) values decrease while engaging in exercise [20]. Cortisol values, as the marker of stress, increase after intensive muscular activity [21,22]. Simultaneous measurement of muscle injury and inflammatory markers is of key importance in clearly defining the potential source of inflammation so that further measures for the athletes’ health can be taken [8].

Calprotectin, as a marker of inflammation released by neutrophils, is not yet widely used in sports medicine, but some studies show that calprotectin is released into the bloodstream during muscle contraction [23]. Research data on the blood levels of calprotectin in athletes are scarce. Mostly, the values were measured after a one-time short and intense or continuous and moderate physical activity, showing the increase in the calprotectin concentrations [24,25]. In our hypothesis, we presume that a progressive increase in the intensity of water polo players’ training affects the concentration of serum calprotectin as well as the other inflammation biochemical markers and muscle injury markers.

This study’s aim was to determine the association of serum calprotectin and the concentration of inflammatory and muscle injury biomarkers with indicators of physical fitness through four phases of dynamically monitoring top athletes during one competitive season at intervals of 10 to 12 weeks. This included the following phases: the beginning of the season, the time of low-intensity training, the time of high-intensity training, and the end of the season. Additionally, we aimed to determine the interrelationship of different biochemical markers and the dynamics of concentration changes in different phases of physical fitness in the competitive season.

## 2. Materials and Methods

### 2.1. Subjects and Experimental Design

Twenty professional water polo male players were enrolled in this study (median age: 21 (15–31)). The participants and parents of minor participants were informed about the experimental procedures and possible discomforts associated with this study before signing a written informed consent. All 20 players completed this study, and the biomarkers measured in this study did not affect the training or strength and conditioning plans of their coaches. In 2020, this study was approved by the Ethical Committee of University Hospital Sveti Duh, Zagreb, Croatia, and conducted according to the Declaration of Helsinki. During the season, the team’s physician regularly checked the health status of all subjects, and all of them were healthy, without chronic or autoimmune diseases.

Experimental procedures were conducted at four (4) time points throughout the whole competitive water polo season: at the beginning of the season (T1), at the time of low-intensity training (T2), at the time of high-intensity training (T3), and at the end of the season (T4). The period between every time point was 10–12 weeks. During each visit, the subjects performed physical tests as markers of physical fitness, which consisted of repetitive strength, maximal strength, and static strength. After a 15 min seated rest, subjects reported to the hospital’s diagnostic laboratory for blood sampling. The sampling occurred every three months, starting in September just before the start of the competitive season and ending in May of the following year. Blood sampling was always carried out at the same time in each phase of the research, thereby reducing daily marker variations to the lowest possible level.

### 2.2. Blood Collection

The blood was collected from the antecubital vein of the dominant hand after an overnight fasting for 12 h, 8 h of sleeping, one day rest, and before any physical examinations, according to the recommendations of the Croatian Society of Medical Biochemistry and Laboratory Medicine [26]. The blood was drawn into 10 mL serum tubes (Becton Dickinson, Franklin Lakes, NJ, USA) with a clot activator. Prior to the centrifugation at 3000× *g* for 10 min, the tubes were kept at room temperature for 30 min to allow the clotting. The serum was aliquoted into two microtubes and stored at −80 °C until analysis.

### 2.3. Biochemical Marker Analysis

The activity of CK was analyzed on a biochemical analyzer, Beckman Coulter AU 680 (Beckman Coulter, Brea, CA, USA), using the photometric method. Concentrations of calprotectin, CRP, and myoglobin were analyzed on a biochemical analyzer, Beckman Coulter AU 680 and Beckman Coulter AU 2700Plus (Beckman Coulter, Brea, CA, USA), using the immunoturbidimetric method. Furthermore, concentrations of interleukin 6 (IL-6), troponin I, and cortisol were analyzed on the immunochemical analyzer UniCel DxI 600 (Beckman Coulter, Brea, CA, USA) using the chemiluminescent method (CLIA). Inter-assay variation was eliminated by analyzing all specimens in a single batch using a single instrument, one analyst, and one set of calibrators with the same lot of reagents.

### 2.4. Test of Physical Fitness

Physical fitness was assessed by the tests of repetitive strength, maximal strength, and static strength. The tests were conducted, supervised, and evaluated by the same researcher.

#### 2.4.1. Repetitive Strength

##### Maximum Sit-Ups in 60 Seconds (SU-MR60′)

The subject lay on his back with his hands clasped behind his head while his legs were bent at the knees, as well as at the hip at a 90-degree angle, and fixed at the floor by an assistant. At the starting signal of the meter, the subject raised the trunk to a forward bend. During descent, the subject must touch the mat with his shoulder blades. The task ended after 60 s. The task was performed only once.

##### Pull-Ups until Failure (PU-MR)

The subject climbed onto the chair and grabbed the pull-up bar with hands shoulder-width apart. The body and the subject’s arms and legs were stretched. From the starting position, the subject rose by bending his arms at the elbows so that his chin reached the height of the bar. The body remained vertical during the performance. The subject’s task was to perform pull-ups correctly as many times as possible. The task was performed only once for each testing phase.

##### Bench Press with 70% of Own Body Weight (BP70%MR)

The examiner set a load of 70% of the subject’s weight. The subject took a proper position on the bench and started lifting weights with proper amplitude and technique. The subject’s task was to perform as many correct repetitions as possible. During heavy load, lifting assistance was required.

##### Squats to 90 Degrees in 60 Seconds (BSQ90°-60′)

The subject stood in a straddling position (slightly wider than the width of the hips) in front of the bench, which was set up earlier so that the subject could descend up to 90 degrees at the hips and knees. Upon the examiner’s sign, the subject started with an even descent into a squat so that he touched the bench with his glutes and then lifted himself up to fully extended legs. The task ended after 60 s. The task was performed only once over the course of testing.

#### 2.4.2. Maximal Strength

##### One Maximal Repetition of Bench Press (BP-1RM)

By gradually lifting the load through 3–6 series, the subject is brought to the situation in which he pushed his maximum weight from a flat bench in one repetition. The “pyramid” work system was used on this occasion, where the number of repetitions was reduced as the weight of the weights increased. During heavy loads, assistance was required, so two of the assistants held the ends of the weights. The resting period between attempts was five minutes.

##### One Squat with Maximal Load (BSQ-1RM)

The subject stood in a straddling position (slightly wider than the width of the hips) between the racks and gripped the bar, making sure that there was an equal distance from the left and right sides to the weight plates. He pulled his head under the bar and stood up with the weight on his back while making sure that his back was straight and flat. The subject lowered himself into a squat at an even pace so that he touched the bench with his glutes and lifted weights to fully extended legs. It started with a load of 60 kg, and each new attempt increased the load by 10 kg. The “pyramid” work system was used on this occasion. In the event of a heavy load, assistance was necessary, so the two assistants, one located behind the subject, preventing him from falling backward, helped with the ends of the weights. The task was carried out until the subject could no longer rise from the squat.

##### One Wide Pull behind the Head (LPU-1RM)

The subject took his position on the lat machine by putting his knees under the stop, which held them so that the weight did not lift them when pulling. He put his hands on the lever in a wide position, and he had to use the power of his back and hand muscles to lower the lever to the height of his ears. By gradually raising the load through 3–6 sets, the subject was brought to a situation where he pulled his maximum weight in one rep. The “pyramid” work system was used on this occasion. In the event of a heavy load, assistance was necessary.

#### 2.4.3. Static Strength

##### Back Extension Test (BET)

The subject lay down with his chest facing the bench so that his superior iliac spine was on the very edge of the bench, his arms in circle and legs extended. The subject raised the trunk until it assumed a horizontal position and then maintained this position. The examiner turned on the stopwatch at the moment when the subject “fixed” the given position. The stopwatch was turned off when the subject disturbed the given position by more than 5 cm. The task was performed only once over the course of testing.

#### 2.4.4. Pain

Pain was evaluated by using Visual Analogue Scale (VAS), with zero (0) meaning no pain and ten (10) meaning the worst possible pain.

### 2.5. Statistical Analysis

Data were analyzed by using SPSS software (IBM SPSS Statistics for Windows, version 24.0. Armonk, NY: IBM Corp., United States). The Shapiro–Wilk test was used to test the normality of the distribution. Since the number of subjects was fewer than 30 and the majority of the data were not normally distributed, the data were expressed as a median, and interquartile range (IQR) and non-parametric statistics were performed. Friedman ANOVA test, with Bonferroni correction for multiple comparisons, was used to test for differences between repeated measures data. Spearman rank correlation test was performed to test the association between variables. A *p*-value ≤ 0.05 was considered statistically significant.

## 3. Results

### 3.1. Biochemical Parameters and Physical Activity Measurements in Four Phases during One Competitive Season

Table 1 presents median values and IQRs for different biochemical parameters and indicators of physical fitness. The Friedman ANOVA test, with repeated measurements, shows that three biochemical markers change statistically significantly over time: calprotectin, cortisol, and CK.

**Figure 1:**
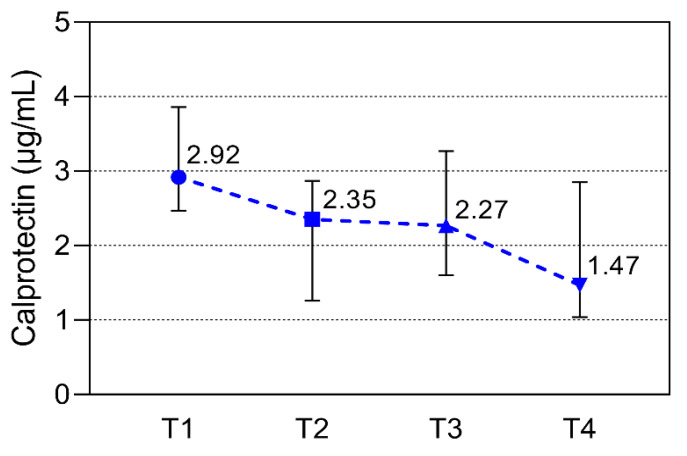
Calprotectin values over the study period at the beginning of the season (T1), at the time of low-intensity training (T2), at the time of high-intensity training (T3), and at the end of the season (T4). The period between every time point was 10–12 weeks. All data are expressed as median and interquartile ranges. Significant (*p* < 0.05) from the corresponding T2 vs. T1 and T4 vs. T1.

**Figure 2:**
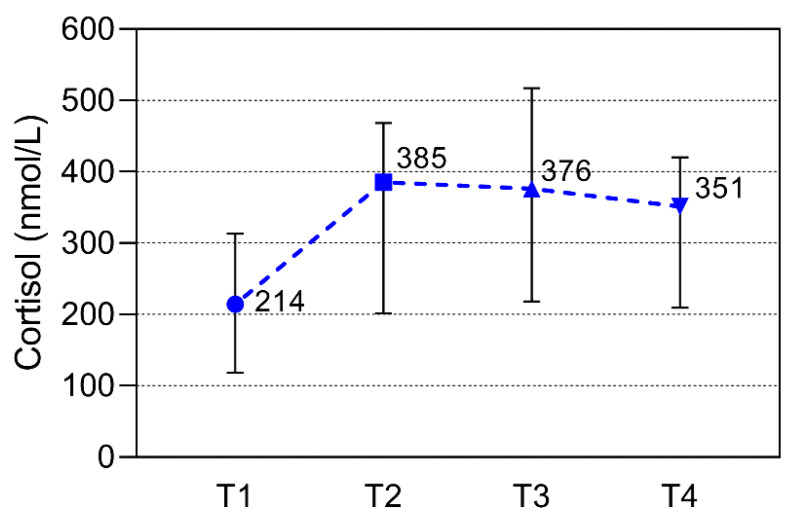
Cortisol values over the study period at the beginning of the season (T1), at the time of low-intensity training (T2), at the time of high-intensity training (T3), and at the end of the season (T4). The period between every time point was 10–12 weeks. All data are expressed as median and interquartile ranges. Significant (*p* < 0.05) from the corresponding T2 vs. T1.

**Figure 3:**
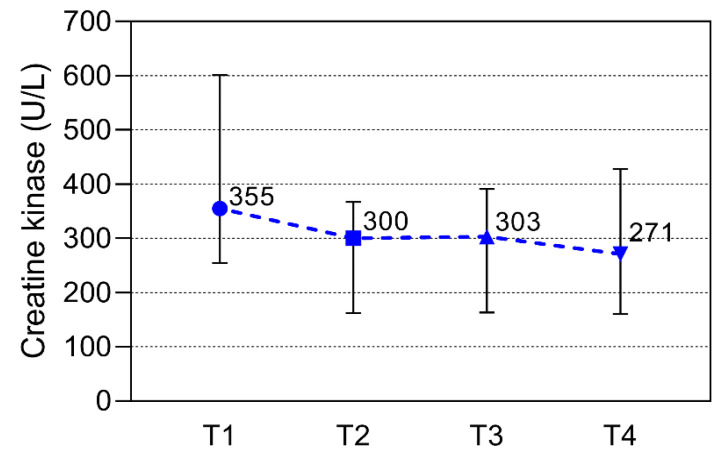
Creatine kinase values over the study period at the beginning of the season (T1), at the time of low-intensity training (T2), at the time of high-intensity training (T3), and at the end of the season (T4). The period between every time point was 10–12 weeks. All data are expressed as median and interquartile ranges. Significant (*p* < 0.05) from the corresponding T2 vs. T1, T3 vs. T1, and T4 vs. T1.

Calprotectin median values significantly differed during the season. There is a significant decrease in calprotectin in the second measurement compared to the first (*p* = 0.004), and the same decrease was observed between the third and fourth measurements (*p* = 0.004), while there were no significant differences between the second and third measurement points (Figure 1). Cortisol median values differed during the season, with a significant increase in cortisol in the second measurement compared to the first one (*p* = 0.011), after which there was no significant difference between the other measurement points (Figure 2). CK median values differ during the season, with a significant decrease in the second, third, and fourth measurements, but only when compared to the first measurement (*p* = 0.003, 0.008, 0.005, respectively). However, there were no statistically significant differences among other monitoring points (Figure 3).

Statistically significant changes were found for all indicators of physical fitness (Table 1). All parameters showed a significant decrease and significant differences among each measurement (*p* < 0.001 for any comparison).

### 3.2. Correlation Analyses between Calprotectin and Other Biochemical Parameters during Different Phases

Spearman rank correlation testing (Table 2) showed that there was no statistically significant difference between calprotectin and other parameters for the first measurement (T1) before the beginning of the competitive season. In the second measurement (T2), a moderate positive correlation was found between calprotectin and hsCRP (*ρ* = 0.47, *p* = 0.043). In the third measurement, calprotectin was moderately positively correlated with hsIL-6 (*ρ* = 0.56, *p* = 0.040) and myoglobin (*ρ* = 0.587, *p* = 0.007). In the fourth measurement, calprotectin positively correlated with cortisol (*ρ* = 0.69, *p* < 0.001) and myoglobin (*ρ* = 0.84, *p* < 0.001).

### 3.3. Correlation Coefficients of Biochemical Parameters with Fitness Indicators

Spearman correlation coefficients (rho, *ρ*) of some biochemical parameters with fitness indicators are presented in Table 3. In the first phase, calprotectin showed a moderately negative correlation with the number of PU-MR (*p* = 0.038) and the number of BSQ90°-60′ (*p* = 0.039), while hsCRP had a moderate positive correlation with BSQ-1RM (*p* = 0.016).

In the second-phase measurements, there were no correlations between calprotectin and physical fitness measurements. Cortisol shows a moderate to high positive correlation with most of the fitness indicators: SU-MR 0′ (*p* = 0.004), number of PU-MR (*p* = 0.020), BP70%MR (*p* = 0.029), number of BSQ90°-60′ (*p* = 0.042), and BP-1RM (*p* = 0.019). There were also moderate positive correlations between hsCRP and BP70%MR (*p* = 0.047).

In the third phase, there were also no correlations between calprotectin and physical fitness measurements, but there was a moderate to high positive correlation of cortisol with SU MR 60′ (*p* = 0.006), BP-1RM (*p* = 0.004), and LPU-1RM (*p* = 0.050). hsIL-6 showed correlation with BP 1RM (*p* = 0.029) and LPU-1RM (*p* = 0.021). Myoglobin showed a moderate to strong correlation with most of the fitness indicators: SU-MR 60′ (*p* = 0.019), PU MR (*p* = 0.010), BP70%MR (*p* = 0.013), BP-1RM, (*p* < 0.001), BSQ 1RM, (*p* = 0.025), and LPU-1RM (*p* = 0.036).

In the fourth phase, calprotectin showed a moderate positive correlation with SU-MR60′ (*p* = 0.029), BP-1RM (*p* = 0.017), and BET (*p* = 0.045). Cortisol showed a moderate to high positive correlation with SU-MR 60′ (*p* = 0.002), PU-MR (*p* = 0.007), BSQ 90° 60′ (*p* = 0.010), and BP-1RM (*p* = 0.002). hsIL-6 showed a moderate to good correlation with SU-MR60′ (*p* = 0.042), squats in 60′ (*p* = 0.041), and BET (*p* = 0.014). Myoglobin showed a moderate to good correlation with the following fitness indicators: SU-MR60′ (*p* = 0.004), PU-MR (*p* = 0.029), BP-1RM (*p* = 0.007), and BET (*p* = 0.016).

## 4. Discussion

Our objective was to investigate the associations between biochemical parameters and the dynamics of concentration change during a water polo player’s competitive season, which is divided into four phases. We also looked at the associations between serum calprotectin and physical markers, inflammation, and muscle injury biomarkers. We observed significant changes in serum calprotectin concentrations over one competitive season, as well as in cortisol concentrations and CK activity. All the physical activity parameters that were examined also exhibited a significant decrease during the season. In some phases of the examination during the season, calprotectin serum concentration correlated with parameters of muscular damage and inflammation as well as with parameters of physical activity. At the beginning of the season, calprotectin was negatively correlated with the parameters of physical activity, while it exhibited a positive correlation with these parameters at the end of the season.

It is well known that moderate physical activity over a longer period may have effects on immune function, and not necessarily positive ones [27]. Although, based on the available literature (there is no research on calprotectin in professional athletes), it has been shown that calprotectin is released during exercise, which includes cycling or long-distance running [23,24,25]. The expression of two different units of calprotectin was increased in skeletal muscle tissue during the exercise [23]. Fagerhol et al. observed that long-distance running induces multiple increases in calprotectin blood concentration [24]. The same study also showed that the increase depends on exercise intensity (i.e., it is the highest after a marathon, medium for a half-marathon, and the lowest in short-term maximal physical exercise until exhaustion) [24]. Additionally, in a study performed on nine healthy male volunteers involved in continuous moderate-intensity exercise (CME) and high-intensity interval exercise (HIIE), Fico et al. showed that lower concentrations of calprotectin were observed following acute HIIE compared to CME [25]. The design of our study included a long-term follow-up in which we observed a progressive decrease in calprotectin concentrations, determined after overnight fasting and rest, at four time points during one competitive season. In the first phase, just before starting the competitive season, the correlation of calprotectin with fitness indicators was negative, indicating that calprotectin concentrations were higher in those participants who had lower values of some fitness indicators, and this might be comparable with the study of Fico at al [25]. The positive correlation with the decrease of fitness indicators at the end, determined in the fourth phase of the season, may be comparable with the study of Fagerhol et al. [24]. However, to the best of our knowledge, there is no study with a similar design that examined the cumulative effect of intensive physical activity in professional sportsmen during one competitive season regarding the values of calprotectin in blood. It seems that regular physical activity in professional athletes leads to a decrease in serum concentrations of calprotectin, and as the season progresses, the values of the physical fitness parameter also decrease due to muscle fatigue. Therefore, in the fourth, most intensive phase after long-term training, the correlation between fitness indicators and protein concentration is significant, and the correlation coefficients are positive.

In our study, CRP, as a marker of inflammation, and IL-6 did not show a statistical difference among four seasonal measurements. However, a decreasing trend of CRP can be noticed. Studies investigating biochemical markers after overnight fasting and rest in soccer players performing preseason low aerobic physical activity showed an increase in CRP values after the preseason training [28,29]. The study showed that training leads to greater inflammation at the beginning of the season than at the end, while some studies that followed athletes (soccer and basketball players) over a longer period of time showed an increase in CRP values [30,31,32]. These results, which are in contrast with our results, show that the type of sport and physical activity may have a role in the physiological response and secretion of certain markers of inflammation, such as CRP. Two studies performed on handball and wrestling sportsmen with a similar study design showed similar results regarding IL-6 concentrations, having peaked in the middle of the season compared to the beginning and the end of the season [33,34]. These results may imply that the beginning of the season and training after a certain period of rest leads to severe inflammation in athletes compared to the end of the season when the human body adapts to the training.

In this study, we showed that cortisol concentrations were higher at the end of the season compared to the beginning of the season, peaking at the second time point, showing that constant training produces certain stress to the organism. A 4-year follow-up study in elite basketball players, which analyzed monthly hormonal responses in relation to playing position and playing time, also showed a progressive increase in cortisol concentrations throughout the season, which is similar to our results [35]. On the other hand, another study in basketball showed that cortisol reaches its peak after preseason training and at the beginning of the second mid-season, with lower values at the beginning and at the end of the season [36]. The increase in cortisol concentration in the third measurement of this study coincides with the basketball tournament, which is certainly an additional stress on physical and psychological bases. Therefore, the cortisol peak is somewhat expected. Similar results to ours were found in a study that monitored American football players from the start of preseason to the end of the competitive college season, which lasted for 12 weeks [37]. A study on European football players showed that cortisol concentrations decrease during the competitive season [30], while in a shorter period (3 months), they increase at the beginning and remain constant until the end [38]. We presume that the observed differences are related to sport-specific training, daily rest, sleeping, differences in the study design and timing of sample collection, as well as the other factors that may affect cortisol excretion. Hence, further research is needed.

A study conducted in water polo players before and after a professional match showed an increase in concentrations of myoglobin and CK, while concentrations of troponin remained constant [39]. In our study, we confirmed that troponin remains constant, while CK decreases during the competitive season and during the period of high-volume training intensity. Although the difference in myoglobin concentrations did not show statistical significance, observed values at the beginning of the competitive season (T1 and T2) were higher than those in the second part (T3 and T4). These results showed that muscles probably adapt to training intensity during the season. Our results are not so similar to the results of the other studies. Thus, studies on athletes from American and European football showed the highest concentrations of muscle damage markers when measured after the preparatory period or in the middle of the competitive season, with a subsequent decrease in values [30,40]. These differences in results probably have a basis in the intensity of training, the schedule of matches, the type of sports activity, etc. Also, blood was sampled 72 h after an official match when the organism would certainly be more exhausted compared to ordinary training [30].

Our study presents some limitations. We only analyzed four time points during one competitive season, and we did not analyze the players’ nutritional status. Additionally, we performed phlebotomy early in the morning, after an overnight rest, which led to the removal of calprotectin from the bloodstream several hours after the training. However, this study also exhibits an additional value due to a specific design where we studied cumulative effects on the changes in biochemical parameters during one competitive season. It is obvious that calprotectin values decrease during the competitive season and with the increasing intensity of training and competition, and the values of physical activity parameters decrease. We would add the evidence indicating a significant decrease in CK activity and an increase in cortisol concentration during the seasonal phases. As a practical application of this study, our results showed that the competitive season in water polo players significantly affects certain biochemical markers, which is why their measurement can positively affect sports performance and the results of sports teams. We anticipate that these findings will enhance athletes’ sports performance and regeneration while preventing overtraining and potential injuries.

## 5. Conclusions

In conclusion, this study showed that the increase in the intensity of water polo players’ training leads to the decrease of calprotectin concentrations determined after an overnight rest during the competitive season, and therefore, it may be considered a marker of physical fitness. We also confirmed the interrelationship between muscle injury markers and inflammatory markers with serum calprotectin, as well as with indicators of physical fitness, through four phases of dynamic monitoring of top athletes during one competitive season.

## Figures and Tables

**Table 1:** Main characteristics of study subjects, including biochemical parameters and physical activity measurements, in four phases during one competitive season.

Biochemical Marker	Median I (25th; 75th)	Median II (25th; 75th)	Median III (25th; 75th)	Median IV (25th; 75th)	*p*-Value
Calprotectin (µg/mL)	2.92 (2.47; 3.86)	2.35 (1.26; 2.87)	2.27 (1.60; 3.27)	1.47 (1.04; 2.85)	0.004
Troponin I, (ng/L)	5.00 (3.25; 9.75)	5.00 (4.00; 8.50)	4.00 (3.00; 8.75)	4.00 (2.25; 10.5)	0.671
Cortisol (nmol/L)	214 (118; 313)	385 (201; 468)	376 (218; 517)	351 (209; 420)	0.049
hsCRP (mg/L)	0.75 (0.32; 1.88)	0.55 (0.29; 0.78)	0.44 (0.26; 0.74)	0.38 (0.31; 0.54)	0.278
hsIL-6 (pg/mL)	1.87 (1.08; 3.48)	1.34 (0.92; 2.59)	1.96 (0.83; 3.15)	1.51 (0.93; 3.29)	0.513
Creatine kinase (U/L)	355 (254; 601)	300 (162; 367)	303 (163; 391)	271 (160; 428)	0.014
Myoglobin (µg/L)	139 (105; 167)	136 (90; 157)	87.0 (59.3; 172.3)	98 (54; 200)	0.173
Pain (VAS)	0	3.00 (2; 4)	4 (2; 6)	4 (2; 6)	<0.001
SU-MR 60′ (N)	63 (59; 84)	57 (53; 78)	49 (45; 70)	46 (44; 66)	<0.001
PU-MR60′ (N)	11 (9; 15)	9 (7; 13)	7 (5; 11)	6 (4; 9)	<0.001
BP 70% MR	38 (33; 42.3)	34 (30; 38)	29 (25; 33)	26 (22; 32)	<0.001
BSQ 90° 60′	55 (51; 62)	48 (44; 55)	41(37; 48)	39 (35; 44)	<0.001
BP-1RM	100 (90; 115)	95 (85; 110)	89 (79; 104)	86 (77; 100)	<0.001
BSQ-1RM	117 (101; 130)	108 (92; 121)	101 (84; 113)	99 (84; 111)	<0.001
LPU-1RM	105 (95; 115)	99 (89; 109)	91 (81; 101)	87 (77; 98)	<0.001
BET	86 (76; 104)	77 (67; 95)	64 (54; 82)	63 (53; 80)	<0.001

Comparison of continuous variables was carried out using non-parametric Friedman ANOVA test for repeated measurements. Variables are expressed as median and interquartile ranges. VAS—Visual Analog Scale; hsCRP—high-sensitivity C-reactive protein; hsIL-6—high-sensitivity interleukin 6; SU MR 60′—maximum sit-ups in 60 s; PU MR—pull-ups until failure; B70% MR—bench press with 70% of own body weight; BSQ90°-60′—squats to 90 degrees in 60 s; BP-1RM—one maximal repetition of bench press; BSQ 1RM—one squat with maximal load; LPU-1RM—one wide pull behind the head; BET—back extension test.

**Table 2:** Correlation coefficients between calprotectin and other biochemical parameters, separately for each phase during one competitive season.

Phase	T1	T2	T3	T4
	Calprotectin (µg/mL)	Calprotectin (µg/mL)	Calprotectin (µg/mL)	Calprotectin (µg/mL)
Troponin I, (ng/L)	−0.18	0.18	0.37	0.41
Cortisol (nmol/L)	0.41	0.10	0.44	0.69 **
hsCRP (mg/L)	0.05	0.47 *	0.18	−0.27
hsIL-6 (pg/mL)	0.17	0.38	0.56 *	0.22
Creatine kinase (U/L)	0.08	0.10	0.10	0.28
Myoglobin (µg/L)	0.02	−0.09	0.58 **	0.84 **

* *p* < 0.05; ** *p* < 0.01.

**Table 3:** Calprotectin, IL-6, CRP, and myoglobin in correlation with fitness indicators, separately for each phase during one competitive season.

	SU-MR 60′ (N)	PU MR (N)	BP70% MR (N)	BSQ90°-60′ (N)	BP-1RM	BSQ-1RM	LPU-1RM	BET
**Phase T1**	Correlation coefficients							
Calprotectin (µg/mL)	−0.29	−0.47 *	−0.08	−0.47 *	−0.20	−0.24	−0.13	−0.16
Cortisol (nmol/L)	−0.19	−0.37	0.40	−0.05	−0.10	−0.12	−0.18	−0.21
hsCRP (mg/L)	−0.13	−0.03	0.28	0.23	0.26	0.53 *	0.20	−0.16
hsIL-6 (pg/mL)	−0.24	0.01	0.05	0.09	−0.11	0.01	−0.05	−0.30
Myoglobin (µg/L)	0.02	0.01	−0.22	−0.25	0.02	−0.02	−0.14	−0.22
**Phase T2**	Correlation coefficients							
Calprotectin (µg/mL)	−0.16	−0.19	0.40	−0.09	0.20	0.13	0.13	−0.05
Cortisol (nmol/L)	0.61 **	0.52 *	0.49 *	0.46 *	0.52 *	0.40	0.41	0.31
hsCRP (mg/L)	−0.11	−0.16	0.45 *	0.02	0.25	0.34	0.03	−0.11
hsIL-6 (pg/mL)	−0.03	0.21	0.37	0.23	0.34	0.29	0.26	−0.01
Myoglobin (µg/L)	0.25	0.38	0.15	0.09	0.35	0.12	0.26	0.23
**Phase T3**	Correlation coefficients							
Calprotectin (µg/mL)	0.19	0.22	0.29	0.08	0.33	0.12	0.24	0.34
Cortisol (nmol/L)	0.59 **	0.41	0.36	0.15	0.61 **	0.42	0.44 *	0.28
hsCRP (mg/L)	−0.19	−0.37	0.21	−0.20	0.00	0.05	0.01	−0.08
hsIL-6 (pg/mL)	0.33	0.43	0.07	0.09	0.57 *	0.50	0.59 *	0.23
Myoglobin (µg/L)	0.52 *	0.56 *	0.54 *	0.33	0.74 **	0.50 *	0.47 *	0.37
**Phase T4**	Correlation coefficients							
Calprotectin (µg/mL)	0.49 *	0.44	0.30	0.24	0.53 *	0.18	0.33	0.45 *
Cortisol (nmol/L)	0.64 **	0.59 **	0.43	0.56 **	0.64 **	0.38	0.43	0.42
hsCRP (mg/L)	−0.19	−0.19	0.44	0.00	−0.11	0.08	−0.04	−0.18
hsIL-6 (pg/mL)	0.50 *	0.48	0.19	0.51 *	0.19	0.12	0.45	0.60 *
Myoglobin (µg/L)	0.61 **	0.49 *	0.39	0.35	0.58 **	0.33	0.42	0.53 *

* *p* < 0.05; ** *p* < 0.01. SU MR 60′—maximum sit-ups in 60 s; PU MR—pull-ups until failure; B70% MR—bench press with 70% of own body weight; BSQ90°-60′—squats to 90 degrees in 60 s; BP-1RM—one maximal repetition of bench press; BSQ 1RM—one squat with maximal load; LPU-1RM—one wide pull behind the head; BET—back extension test.

## Data Availability

The data that support the findings of this study are available on request from the corresponding author. The data are not publicly available due to privacy or ethical restrictions.
