# Correction: Bukvić et al. The Association of Serum Calprotectin with Fitness Indicators and Biochemical Markers in High-Level Athletes: A Continuous Dynamic Monitoring During One Competitive Season. *Sports* 2023, *11*, 243

**DOI:** 10.3390/sports14020066

**Published:** 2026-02-05

**Authors:** Frane Bukvić, Alan Ivković, Helena Čičak, Lora Dukić, Ana-Maria Šimundić, Domagoj Marijančević, Daria Pašalić

**Affiliations:** 1 Department of Orthopedics and Trauma Surgery, University Hospital ‘Sveti Duh’, 10000 Zagreb, Croatia; frane.bukvic@gmail.com (F.B.); alan.ivkovic@gmail.com (A.I.); 2 School of Medicine, University of Zagreb, 10000 Zagreb, Croatia; 3 Department of Medical Laboratory Diagnostics, University Hospital ‘Sveti Duh’, 10000 Zagreb, Croatia; helena.cicak5@gmail.com (H.Č.); lora.dukic@gmail.com (L.D.); am.simundic@gmail.com (A.-M.Š.); 4 Faculty of Pharmacy and Biochemistry, University of Zagreb, 10000 Zagreb, Croatia; 5 Department of Clinical Chemistry, University Hospital Centre ‘Sestre Milosrdnice’, 10000 Zagreb, Croatia; domagoj.marijancevic@kbcsm.hr; 6 Department of Medical Chemistry, Biochemistry and Clinical Chemistry, School of Medicine, University of Zagreb, 10000 Zagreb, Croatia

**Text Correction**

There was an error in the original publication [1]. After carefully reviewing the published version, we noticed two inaccuracies in the Methods Section. The median age and age range of the participants was incorrectly reported as 28 (22–42), whereas the correct range is 21 (15–31). Therefore, in addition to the statement of signed informed consent, it is necessary to add that for a minor participant, the consent was obtained from a parent. In addition, the storage temperature of the samples was incorrectly stated as −20 °C, while the correct storage condition was −80 °C. These corrections concern the accurate description of the study sample and methodology. They do not affect the results, analysis, discussion, or conclusions of the article.

A correction has been made to the Abstract and Sections 2.1 and 2.2.

Abstract: Twenty professional male water polo players (median age: 21 (15–31)) were included in this study.

2. Materials and Methods

2.1. Subjects and Experimental Design

Twenty professional water polo male players were enrolled in this study (median age: 21 (15–31)). The participants and parents of minor participants were informed about the experimental procedures and possible discomforts associated with this study before signing a written informed consent.

2.2. Blood Collection

The serum was aliquoted into two microtubes and stored at −80 °C until analysis.

**Informed Consent Statement Correction**

The Informed Consent Statement has been corrected.

Informed Consent Statement: Informed consent was obtained from all subjects and their guardians involved in the study.

The authors state that the scientific conclusions are unaffected. This correction was approved by the Academic Editor. The original publication has also been updated.

## References

[1] Bukvić F., Ivković A., Čičak H., Dukić L., Šimundić A.-M., Marijančević D., Pašalić D. (2023). The Association of Serum Calprotectin with Fitness Indicators and Biochemical Markers in High-Level Athletes: A Continuous Dynamic Monitoring during One Competitive Season. Sports.

